# Ge N-Channel MOSFETs with ZrO_2_ Dielectric Achieving Improved Mobility

**DOI:** 10.1186/s11671-021-03577-0

**Published:** 2021-08-04

**Authors:** Lulu Chou, Yan Liu, Yang Xu, Yue Peng, Huan Liu, Xiao Yu, Genquan Han, Yue Hao

**Affiliations:** 1 State Key Discipline Laboratory of Wide Band Gap Semiconductor Technology, School of Microelectronics, Xidian University, Xi’an, 710071 People’s Republic of China; 2 Intelligent Chip Research Center, Zhejiang Lab, Hangzhou, 311121 People’s Republic of China

**Keywords:** Germanium, ZrO_2_, MOSFET, CMOS, Mobility

## Abstract

High-mobility Ge nMOSFETs with ZrO_2_ gate dielectric are demonstrated and compared against transistors with different interfacial properties of ozone (O_3_) treatment, O_3_ post-treatment and without O_3_ treatment. It is found that with O_3_ treatment, the Ge nMOSFETs with ZrO_2_ dielectric having a EOT of 0.83 nm obtain a peak effective electron mobility (*μ*_eff_) of 682 cm^2^/Vs, which is higher than that of the Si universal mobility at the medium inversion charge density (*Q*_inv_). On the other hand, the O_3_ post-treatment with Al_2_O_3_ interfacial layer can provide dramatically enhanced-*μ*_eff_, achieving about 50% *μ*_eff_ improvement as compared to the Si universal mobility at medium *Q*_inv_ of 5 × 10^12^ cm^−2^. These results indicate the potential utilization of ZrO_2_ dielectric in high-performance Ge nMOSFETs.

## Background

GERMANIUM (Ge) has exhibited advantages of higher carrier mobility and lower processing temperature compared with Si devices. These make Ge to be an alternative for applications of ultrascaled CMOS logic devices and thin-film transistors (TFTs) as top layer in three-dimensional integrated circuits [1–3]. In the past few years, great efforts have been focused on surface passivation, gate dielectric, and channel engineering for Ge p-channel metal–oxide–semiconductor field-effect transistors (MOSFETs), which have contributed to significant improvement in electrical performance for the p-channel devices.

But for Ge n-channel MOSFETs, low effective carrier mobility (*μ*_eff_) caused by poor interfacial layer of gate stack strongly limits the performance of the devices. Various surface passivation techniques including Si passivation [1], plasma post-oxidation [4], and InAlP passivation [5] and several high-κ dielectrics including HfO_2_, ZrO_2_ [6–8], Y_2_O_3_ [9], and La_2_O_3_ [10] have been explored in Ge nMOSFETs to boost the electron *μ*_eff_. It was demonstrated that ZrO_2_ dielectric integrated with Ge channel can provide a robust interface due to that a GeO_2_ interfacial layer can react and intermix with the ZrO_2_ layer [7]. A decent hole *μ*_eff_ has been reported in Ge p-channel transistors [6–8], while there is still a lot of room for improvement in electron *μ*_eff_ for their counterparts.

In this work, Ge nMOSFETs with ZrO_2_ gate dielectric are fabricated to achieve improved *μ*_eff_ over Si in the entire range of inversion charge density (*Q*_inv_). Ge transistors obtain a 50% improvement in electron *μ*_eff_ compared to the Si universal mobility at a medium *Q*_inv_ of 5.0 × 10^12^ cm^−2^.

## Experimental

**Fig. 1:**
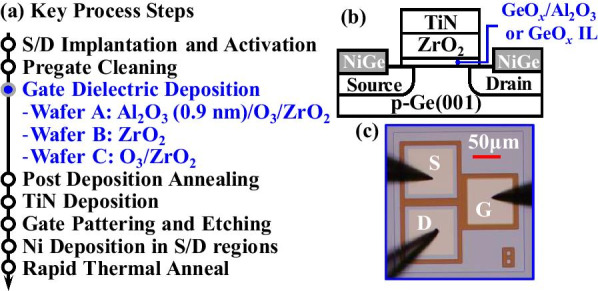
**a** Key process steps for fabricating Ge nMOSFETs. **b** Cross-sectional schematic and **c** microscope image of the fabricated devices

The key process steps for fabricating Ge nMOSFETs on 4-inch p-Ge(001) wafers with a resistivity of 0.136–0.182 Ω cm are shown in Fig. 1a. The source/drain (S/D) regions were implanted with phosphorous ion at a dose of 1 × 10^15^ cm^−2^ and an energy of 30 keV followed by dopant activation annealing at 600 °C. After the pre-gate cleaning, Ge wafers were loaded into an atomic layer deposition chamber for the formation of the gate dielectric layer(s): Al_2_O_3_/O_3_ oxidation/ZrO_2_, ZrO_2_, or O_3_ oxidation/ZrO_2_ for wafers A, B, or C, respectively. For wafer A, 0.9 nm Al_2_O_3_ was used to protect the channel surface during O_3_ oxidation. O_3_ oxidation was carried out at 300 °C for 15 min for both wafers A and C. For all the wafers, the thickness of ZrO_2_ was ~ 3.3 nm. Subsequently, TiN(100 nm) gate metal was deposited via physical reactive sputtering, and lithography patterning and reactive ion etching were used to form the gate electrode. After that, a 25-nm-thick Ni layer was deposited in S/D regions. Finally, the post-metallization annealing (PMA) at 350 °C for 30 s was carried out to form the Ni germanide and improve the interface quality. Schematic and microscope images of the fabricated transistor are shown in Fig. 1b, c, respectively.

**Fig. 2:**
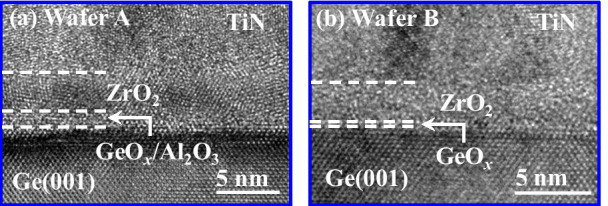
HRTEM images of **a** TiN//ZrO_2_/Al_2_O_3_/GeO_*x*_/Ge, **b** TiN/ ZrO_2_/GeO_*x*_/Ge stacks for the devices on wafers A and B, respectively

Figure 2a, b shows the high-resolution transmission electron microscope (HRTEM) images of the gate stacks on wafers A and B, respectively. The unified thickness of the Al_2_O_3_/GeO_*x*_ interfacial layer (IL) for wafer A is ~ 1.2 nm indicating the 0.2–0.3 nm GeO_*x*_. For the device on wafer B, an ultrathin GeO_*x*_ IL was experimentally demonstrated [7].

## Results and Discussion

**Fig. 3:**
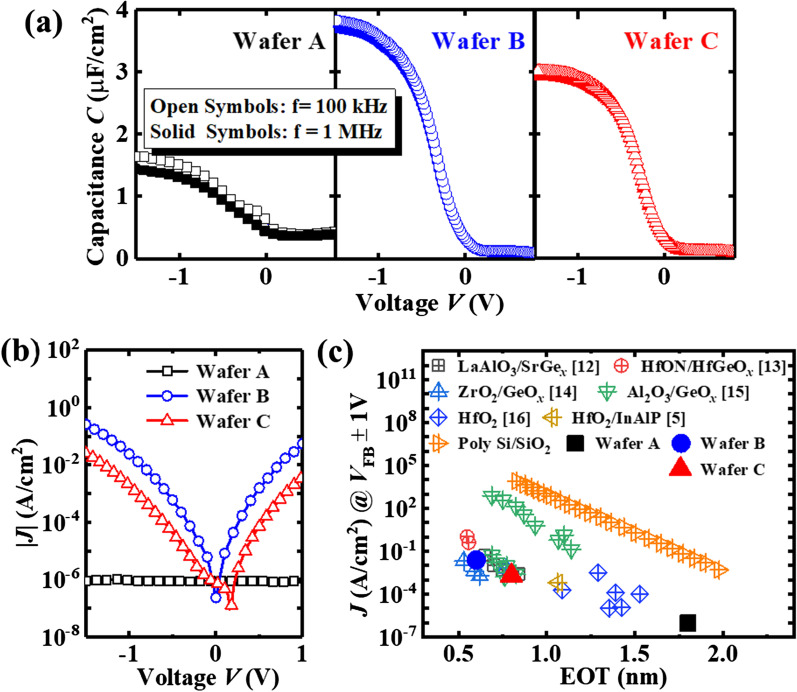
**a** Measured *C* as a function of voltage *V* characteristics for Ge pMOS capacitors on wafers A, B, and C. **b** *J* versus *V* curves for the devices. **c** Benchmarking of *J* (extracted at *V*_FB_ ± 1 V) of the Ge MOS capacitors in this work against data obtained for similar bias conditions from the literature

The measured capacitance (*C*) and the leakage current (*J*) characteristics for Ge MOS capacitors on wafers A, B, and C are measured and shown in Fig. 3a, b, respectively. The equivalent oxide thickness (EOT) of the devices on wafers A, B, and C is extracted to be 1.79, 0.59, and 0.83 nm, respectively. Assuming the GeO_*x*_ IL provides an extra EOT of ~ 0.25 nm for wafers A and C by comparing wafers B and C, the 3.3 nm ZrO_2_ contributes an EOT of ~ 0.6 nm with κ value of ~ 21.8, which is consistent with the previous reported value of amorphous ZrO_2_ [11].These derived results also confirm that the thickness in GeO_*x*_ IL on wafer B is negligible.

The GeO_*x*_/Al_2_O_3_ IL for wafer A and GeO_*x*_ IL for wafer C produces the EOT of ~ 1.2 and ~ 0.25 nm, respectively. The EOT of the devices can be further reduced by decreasing the IL thickness or improving the interface quality, and enhancing the permittivity of ZrO_2_ with some surface passivation, e.g., NH_3_/H_2_ plasma treatment [6]. Figure 3c compares *J* versus EOT characteristics for the Ge nMOSFETs in this work against values for other reported Ge devices [5, 12–17]. It is also observed that the results are consistent with the reported Ge MOS with ultra-thin EOT following the same trends, indicating the difference of leakage current shown in Fig. 3b should be mainly attributable to the difference of EOT.

**Fig. 4:**
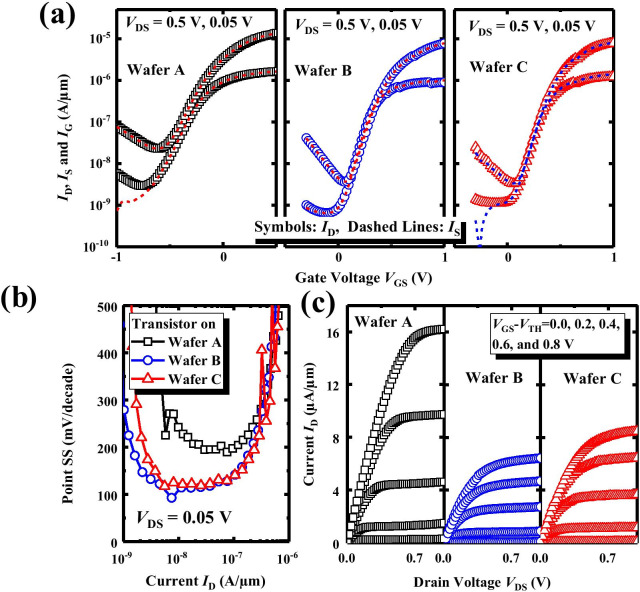
**a** Measured *I*_D_ and *I*_S_ versus *V*_GS_ curves of Ge nMOSFETs on wafers A, B, and C. **b** Point SS as a function of *I*_D_ for the transistors. **c** *I*_D_–*V*_D_ characteristics show that the Ge nMOSFET on wafer A has a higher drive current compared to the devices on wafers B and C

Figure 4a shows measured drain current (*I*_D_) and source current (*I*_S_) versus gate voltage (*V*_G_) curves of Ge nMOSFETs from wafers A, B, and C. All transistors have a gate length *L*_G_ of 4 μm and a gate width *W* of 100 μm. The point subthreshold swing (SS), defined as d*V*_G_/d(log*I*_D_), as a function of *I*_D_ curves for the transistors in Fig. 4a is calculated and shown in Fig. 4b. It is clarified that the transistor on wafer A exhibits the degraded *I*_D_ leakage floor and SS compared to the devices on wafers B and C. Besides the increase in EOT in devices on wafer A would bring in the increment of SS, these phenomenon should be partly attributed to the fact that the device with the Al_2_O_3_ inserted layer has a higher density of interface traps (*D*_it_) within the bandgap of the Ge channel in comparison with the wafers B and C.

Figure 4c shows the measured output characteristics, i.e.,* I*_D_–*V*_D_ curves for various values of gate overdrive |*V*_G_–*V*_TH_| of the devices demonstrating that the Ge transistor on wafer A achieves significantly improved drive current compared to the devices on wafers B and C. Here, *V*_TH_ is defined as *V*_GS_ corresponding to an *I*_D_ of 10^−7^ A/μm. Considering the identical conditions for S/D formation, the boosted *I*_DS_ for transistors on wafer A indicates the higher *μ*_eff_ [18–21]. The Al_2_O_3_ layer has not led to the degradation of *D*_it_ performance near the conduction band of the Ge channel.

**Fig. 5:**
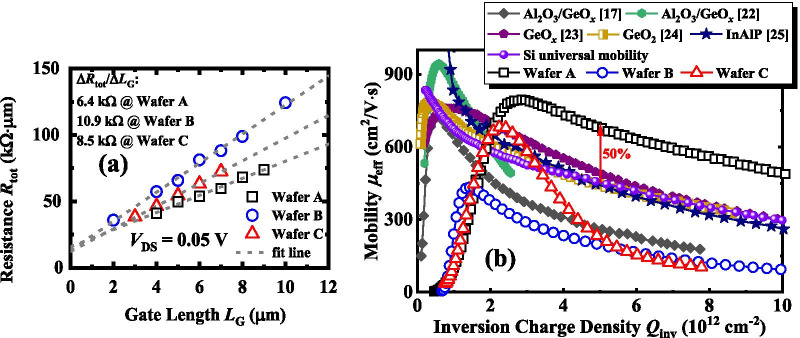
**a** *R*_tot_ versus *L*_G_ curves for Ge nMOSFETs on wafers A, B, and C. The fitted line intersecting at the y-axis and the slope of linear fit lines are utilized to extract the *R*_SD_ and *R*_CH_, respectively. **b** *μ*_eff_ for the Ge nMOSFETs in this work versus previously published results for unstrained Ge transistors. The devices on wafer A show the improved *μ*_eff_ than the Si universal mobility in the entire range of *Q*_inv_

Figure 5a shows the total resistance *R*_tot_ as a function of *L*_G_ for the Ge nMOSFETs with ZrO_2_ dielectric with an *L*_G_ ranging from 2 to 10 µm. The values of *R*_tot_ are extracted at a gate overdrive of 0. 6 V and a *V*_D_ of 0.05 V. The S/D resistance *R*_SD_ of the transistors is extracted to be ~ 13.5 kΩ μm, utilizing the fitted lines intersecting at the *y*-axis. The similar *R*_SD_ is consistent with the identical process of PMA and S/D formation. The channel resistance *R*_CH_ values of the devices are obtained by the slope of the fitted lines, i.e., Δ*R*_tot_/Δ*L*_G_, which can be used for calculating the *μ*_eff_ characteristics of Ge nMOSFETs. To evaluate the interface quality, interface trap densities (*D*_it_) were extracted by the following equation according to Hill’s method [17]:

$$D_{{{\text{it}}}} = \frac{{2G_{{{\text{m}}\max }} /\omega }}{{qA\left[ {\left( {\frac{{G_{{{\text{mmax}}}} }}{{\omega C_{{{\text{ox}}}} }}} \right) + \left( {1 - C_{{\text{m}}} /C_{{{\text{ox}}}} } \right)^{2} } \right]}}$$

where *q* is the electronic charge, *A* is the area of the capacitor, *G*_m,max_ is the maximum value of measured conductance, with its corresponding capacitance *C*_m_, *ω* is the angular frequency, and *C*_ox_ is gate oxide capacitance. The *D*_it_ values are calculated to be 3.7, 3.2, and 2.3 × 10^12^ eV^−1^ cm^−2^ for the devices on wafers A, B, and C, respectively.

It is known that the calculated values correspond to the midgap *D*_it_. The device with Al_2_O_3_ IL on wafer A has a higher midgap *D*_it_ compared to the devices on wafers B and C. This is consistent with the results in Figs. 3a and 4a, and the higher midgap *D*_it_ gives rise to a larger depletion capacitance dispersion in wafer A causing a higher leakage current of *I*_DS_ in comparison with the other two wafers. Note the wafer A should have the lower *D*_it_ near the conduction bandgap due to its higher *μ*_eff_ over wafers B and C.

It is well known that *μ*_eff_ is the bottleneck for high drive current and transconductance in Ge nMOSFETs. Here, *μ*_eff_ can be calculated by $\mu_{{{\text{eff}}}} = 1/[WQ_{{{\text{inv}}}} (\Delta R_{{{\text{tot}}}} /\Delta L_{{\text{G}}} )]$, where Δ*R*_tot_/Δ*L*_G_ is the slope of the *R*_tot_ versus *L*_G_ as shown in Fig. 5a. *Q*_inv_ can be obtained by integrating the measured *C*_inv_–*V*_G_ curves. In Fig. 5b, we compare the *μ*_eff_ versus *Q*_inv_ of the Ge nMOSFETs on wafers A, B, and C with those reported previously in [18, 22–25]. The extracted peak *μ*_eff_ values of the transistors on wafers A and C are 795 and 682 cm^2^/V s, respectively, and that of Ge nMOSFETs on wafer B is 433 cm^2^/V s. Ge nMOSFETs with Al_2_O_3_ IL achieve a significantly improved *μ*_eff_ in comparison with the transistors on wafer B or C, the devices in [18, 22–25] in a high field, and Si universal mobility in the entire *Q*_inv_ range. At a *Q*_inv_ of 5 × 10^12^ cm^−2^, a 50% *μ*_eff_ enhancement is achieved in devices on wafer A as compared to the Si universal mobility. This demonstrates that by protecting the channel surface for preventing the intermixing of ZrO_2_ and GeO_*x*_ using Al_2_O_3_, a high-quality interface between gate insulator and Ge is realized to boost the mobility characteristics, which is also reported in the previous studies of Ge MOSFETs with ultrathin EOT [26]. *μ*_eff_ in transistors on wafer C is higher than the Si universal at a *Q*_inv_ of 2.5 × 10^12^ cm^−2^, although it rapidly decays with the increase in *Q*_inv_ range. This indicates that the used O_3_ oxidation before ZrO_2_ deposition would improve the interfacial quality to some extent; however, it does not lead to enough flat channel surface to effectively suppress the surface roughness scattering of the carrier at high *Q*_inv_ due to the intermixing of ZrO_2_ and GeO_*x*,_ since it is reported that the generation of oxygen vacancies during the intermixing would increase the short-range order (SRO) roughness [27]. Optimizing the O_3_ oxidation process or reducing the Al_2_O_3_ IL thickness can make the Ge transistor achieve a reduced EOT while maintaining a higher *μ*_eff_ at the high *Q*_inv_.

## Conclusions

The impacts of gate dielectric structure and morphology on Ge nMOSFET electrical characteristics are investigated. An Al_2_O_3_/ZrO_2_ gate dielectric provides for significantly-improved *μ*_eff_ as compared to the Si universal mobility. *μ*_eff_ can be improved by inserting an Al_2_O_3_ layer between the ZrO_2_ and Ge channel, which, however, inevitably leads to a larger EOT. Al_2_O_3_-free Ge nMOSFETs with O_3_ oxidation of the Ge surface prior to ZrO_2_ deposition achieve a peak *μ*_eff_ of 682 cm^2^/V s which is higher than that of Si at the similar *Q*_inv_.

## Data Availability

The datasets supporting the conclusions of this article are included in the article.

## Abbreviations

Ge: Germanium
ZrO_2_: Zirconium dioxide
Al_2_O_3_: Aluminum oxide
O_3_: Ozone
Si: Silicon
PMA: Post-metal annealing
PDA: Post-deposition annealing
IL: Interfacial layer
TiN: Titanium nitride
MOSFETs: Metal–oxide–semiconductor field-effect transistors
ALD: Atomic layer deposition
HF: Hydrofluoric acid
*µ*_eff_: Effective carrier mobility
PPO: Plasma post-oxidation
SS: Subthreshold swing
CET: Capacitance-equivalent thickness
EOT: Equivalent oxide thickness
Qinv: Inversion charge density
HRTEM: High-resolution transmission electron microscope
Ni: Nickel
GeO_*x*_: Germanium oxide
*I*_DS_: Drain current
*V*_GS_: Gate voltage
*V*_TH_: Threshold voltage
